# Comparative analysis of sex chromosomes in *Leporinus* species (Teleostei, Characiformes) using chromosome painting

**DOI:** 10.1186/1471-2156-14-60

**Published:** 2013-07-03

**Authors:** Patrícia Pasquali Parise-Maltempi, Edson Lourenço da Silva, Willem Rens, Frances Dearden, Patricia CM O’Brien, Vladimir Trifonov, Malcolm A Ferguson-Smith

**Affiliations:** 1 Departamento de Biologia, Laboratório de Citogenética, Instituto de Biociências, Universidade Estadual Paulista “Julio de Mesquita Filho” - UNESP, Rio Claro, Av. 24A, 1515, CEP 13506-900 Rio Claro, SP, Brazil; 2 Department of Veterinary Medicine, University of Cambridge, Cambridge Resource Centre for Comparative Genomics, Madingley Road, CB3 0ES Cambridge, UK; 3 Institute of Molecular and Cellular Biology, Siberian Branch of the Russian Academy of Sciences, Novosibirsk, Russia

**Keywords:** FISH, Zoo-FISH, Microdissection, Sex chromosome evolution

## Abstract

**Background:**

The *Leporinus* genus, belonging to the Anostomidae family, is an interesting model for studies of sex chromosome evolution in fish, particularly because of the presence of heteromorphic sex chromosomes only in some species of the genus. In this study we used W chromosome-derived probes in a series of cross species chromosome painting experiments to try to understand events of sex chromosome evolution in this family.

**Results:**

W chromosome painting probes from *Leporinus elongatus*, *L. macrocephalus* and *L. obtusidens* were hybridized to each others chromosomes. The results showed signals along their W chromosomes and the use of *L. elongatus* W probe against *L. macrocephalus* and *L. obtusidens* also showed signals over the Z chromosome. No signals were observed when the later aforementioned probe was used in hybridization procedures against other four Anostomidae species without sex chromosomes.

**Conclusions:**

Our results demonstrate a common origin of sex chromosomes in *L. elongatus*, *L. macrocephalus* and *L. obtusidens* but suggest that the *L. elongatus* chromosome system is at a different evolutionary stage. The absence of signals in the species without differentiated sex chromosomes does not exclude the possibility of cryptic sex chromosomes, but they must contain other *Leporinus* W sequences than those described here.

## Background

The origin and evolution of sex chromosomes have interested evolutionary biologists for a long time. Although sex chromosomes evolve from an autosomal pair [1], over time they become different both in gene content and structure [2]. While sex chromosomes in most mammals are ancient, sex chromosomes in some fish, insects and dioecious plants are evolutionarily young [2,3].

Among vertebrates, fish present an enormous diversity of sex determination mechanisms, contrasting with the much more stable systems found in mammals and birds. The majority of teleost fish are gonochoristic, meaning that they exist as males and females, and the most gonochoristic species have genetic sex determination. Beside simultaneous and sequential hermaphroditism, gonochorism in fish is controlled by many forms of sex determination involving genetic and/or environmental factors, although temperature-dependent sex determination might be rarer than previously thought [4,5]. Despite a large number of fish species described, only a minor fraction has been thoroughly investigated and their sex determination mechanism unequivocally clarified [6].

Within the fish species with genetic sex determination many variations have been found, ranging from male or female heterogamety to polygenic sex determination. Multiple sex chromosomes, or autosomal modifiers that enhance or antagonize the sex-determining genes on the gonosomes, are quite frequent [7,8]. This, together with the fact that even between closely related fish species the sex determination mechanisms can be different [6,9-13], demonstrates the high evolutionary plasticity of this fundamental process [7].

Sex chromosomes in the genus *Leporinus* (Teleostei, Anostomidae) present some features that make this group very interesting for the study of sex determination mechanisms and sex chromosome structure, composition and evolution. In general, the sex chromosomes of the *Leporinus* genus have morphological similarities. An exception is observed in the *Leporinus* sp2 species, where the W chromosomes are not differentiated from autosomes. The *Leporinus* group consists of around 40 species and eight of them show heteromorphic sex chromosomes and the presence of conspicuous large blocks of heterochromatin on sex chromosomes is the characteristic feature of the *Leporinus* karyotypes. Among this eight species seven (*L. conirostris*, *L. elongatus*, *L.* aff. *elongatus*, *L. macrocephalus*, *L. obtusidens*, *L. reinhardti*, *L. trifasciatus*) possess enlarged W chromosomes in comparison to the Z, due the increase of heterochromatin, while one, *Leporinus* sp2, presents a karyotype with the W chromosome equal in size to its homologous Z and the autosomes. The hypothesis of the accumulation of repetitive elements as the major pathway in the differentiation of sex chromosomes in Anostomidae is widely accepted [14,15]. In fact, it has been proposed that repetitive elements are different among some species [16] and that they were probably responsible for the recent origin of a multiple Z_1_Z_1_Z_2_Z_2_/Z_1_W_1_Z_2_W_2_ sex chromosome system in *L. elongatus*[15]. However whether the increase of heterochromatic segments by accumulation of repetitive DNA is the cause or consequence of sex chromosome origin remains, as yet, undiscovered and details of sex chromosome evolution within the genus are unknown.

To study sex chromosome evolution in *Leporinus* and related Anostomidae species we have obtained whole W chromosome specific probes of three *Leporinus* species by manual microdissection and used the paints for cross species hybridisation. Our results provide some new views of Anostomidae chromosome evolution and add new evidence on the origin of sex chromosomes in *Leporinus*.

## Results

### Hybridisation using *Leporinus elongatus W* probe (WLe)

**Figure 1:**
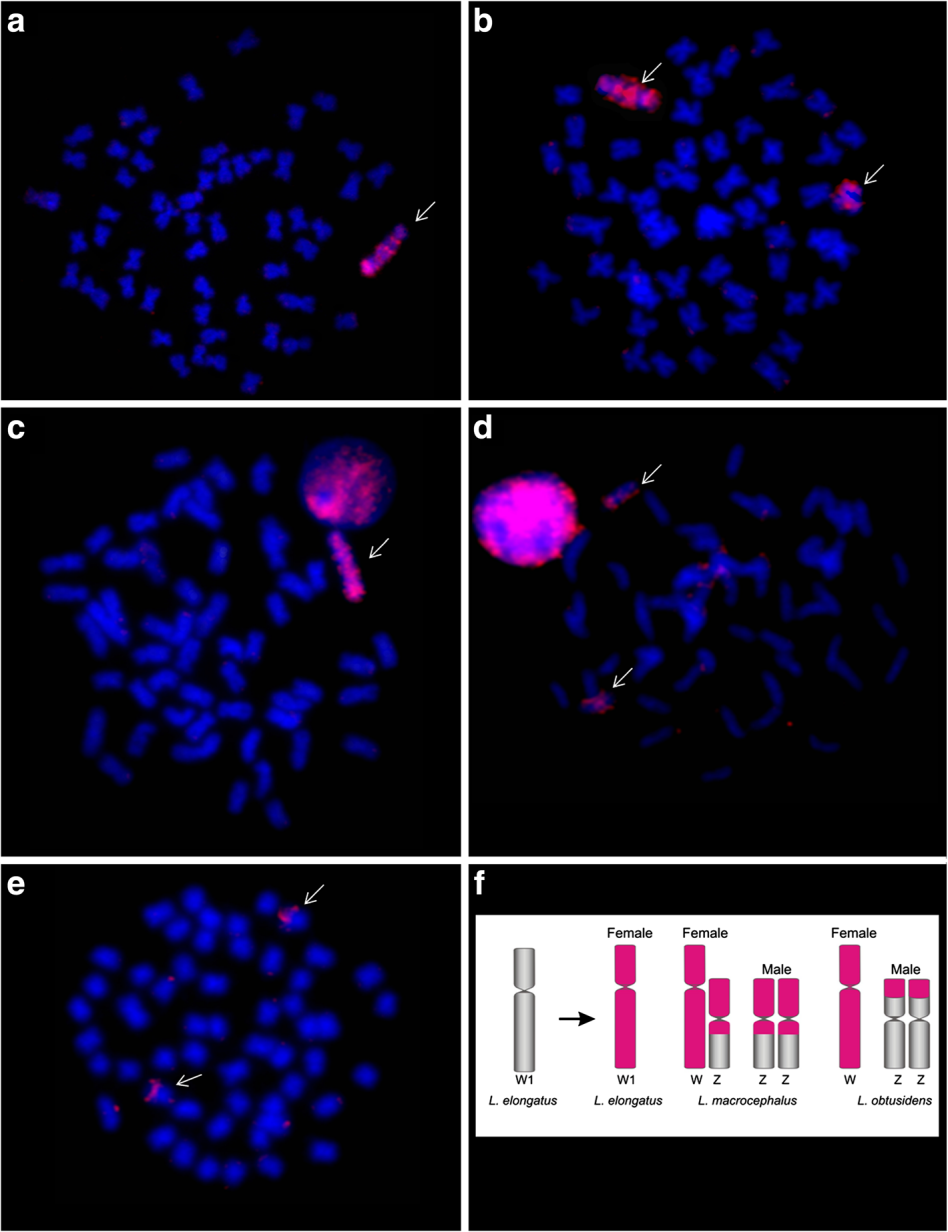
**FISH using *Leporinus elongatus *W chromosome (WLe) probes. a**- WLe vs female *L. elongatus;***b**- WLe vs female *L. macrocephalus;***c**- WLe vs female *L. obtusidens;***d**- WLe vs male *L. macrocephalus;***e**- WLe vs male *L. obtusidens*; **f**- WLe probe hybridisation pattern.

The WLe probe used against chromosomes of the *Leporinus elongatus* female painted the entire W chromosome (Figure 1a). In female individuals of *L. macrocephalus*, the WLe probe painted the entire W chromosome as well as a large part of the Z chromosome (Figure 1b). In male individuals of *L. macrocephalus* the WLe probe painted almost all of both Z chromosomes (Figure 1d). The WLe probe completely painted the W chromosomes of the female *L. obtusidens* (Figure 1c). In *L. obtusidens* males signals were observed in the subtelomeric region of the p-arm of the Z chromosome, but only after increased exposure time (Figure 1e).

FISH experiments with WLe probes in *L. friderici, L. striatus*, *L. lacustris*, *Schizodon borelii* and *S. isognathus* individuals did not produce any result (even under low stringency wash conditions) (data not shown).

### Hybridisation using *Leporinus macrocephalus W* probe (WLm)

**Figure 2:**
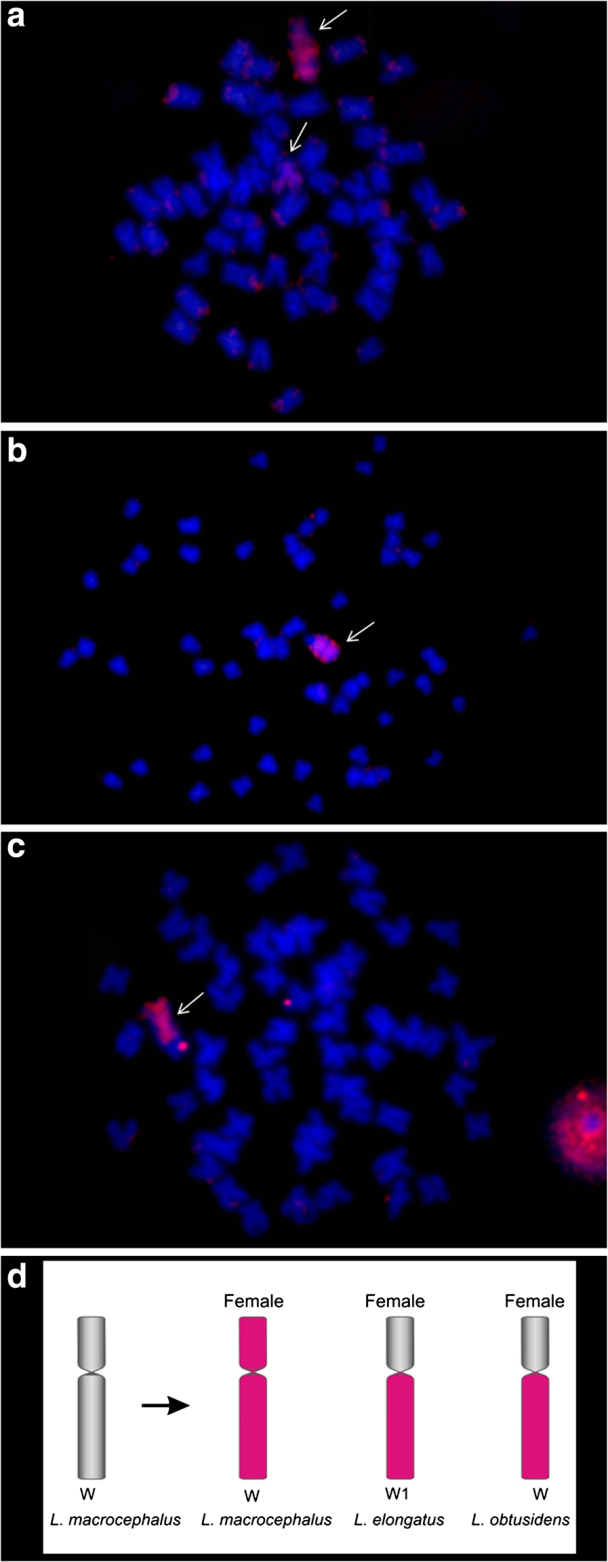
**FISH using *Leporinus macrocephalus *W chromosome (WLm) probes. a**- WLm vs female *L. macrocephalus;***b**- WLm vs female *L. elongatus;***c**- WLm vs female *L. obtusidens*. **d**- WLm probe hybridisation pattern.

FISH experiments using WLm probes showed a hybridisation pattern where the W chromosome of *L. macrocephalus* was entirely painted as well as part of the Z chromosome (Figure 2a). Also, signals on the long arm of the W1 chromosome of *L. elongatus* and in the W chromosome of *L. obtusidens* were observed (Figure 2b, c). In *L. elongatus* male individuals, no hybridisation signals were seen, as in the experiments using the WLe probe onto the *L. elongatus* male.

### Hybridisation using *Leporinus obtusidens W* probe (WLo)

**Figure 3:**
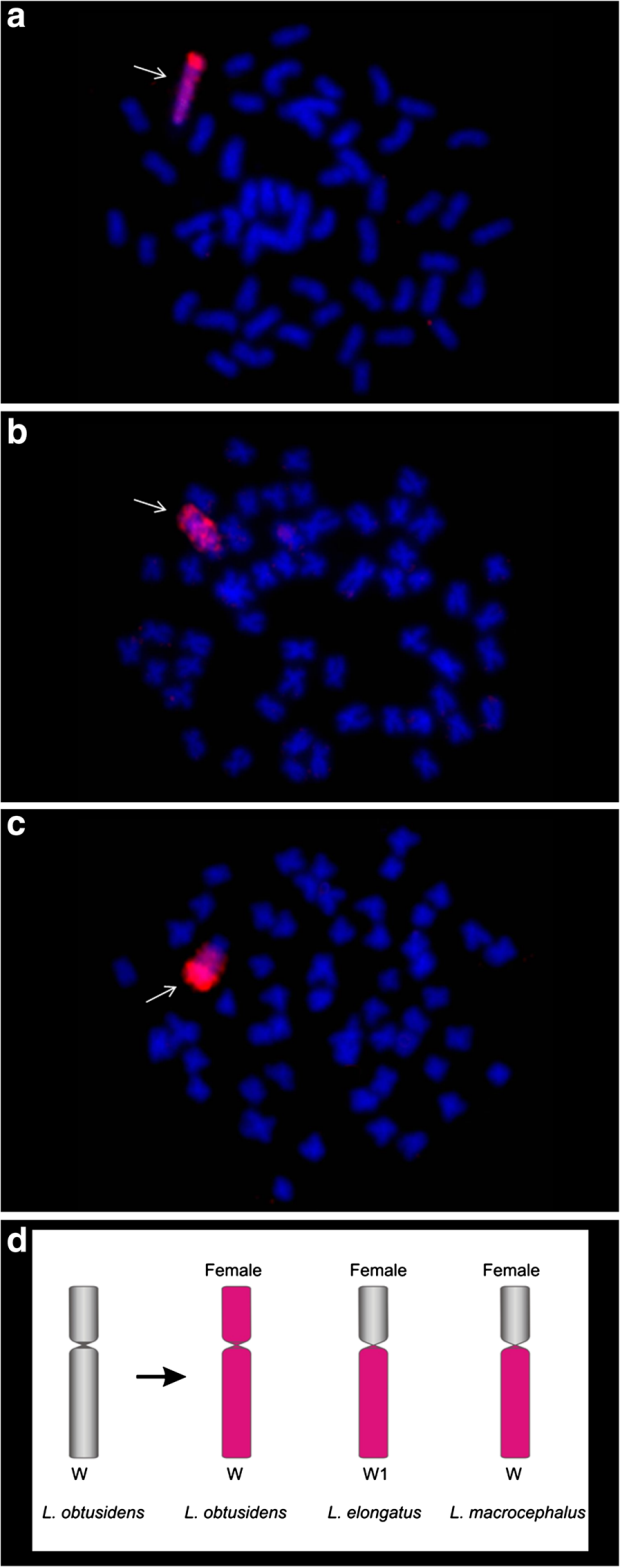
**FISH using *Leporinus obtusidens *W chromosome (WLo) probes. a**- WLo vs female *L. obtusidens;***b**- WLo vs female *L. elongatus;***c**- WLo vs female *L. macrocephalus*; **d**- WLo probe hybridisation pattern.

The WLo probe painted the entire W chromosome of all *L. obtusidens* females (Figure 3a). Signals of similar intensity were also observed in the long arm of W1 chromosomes of *L. elongatus* and W of *L. macrocephalus*, while signals were absent on the short arm (Figure 3b, c). As it was observed in experiments using W from *L. macrocephalus*, no signals were detected in male individuals of all analysed species.

## Discussion and conclusion

The W chromosome of *Leporinus* species is easily recognised, being the largest chromosome of the karyotype due to the accumulation of large heterochromatic blocks, which facilitate the identification of the chromosome during microdissection procedures. While the sex chromosomes of the *Leporinus* species in general are highly differentiated, the other chromosomes show the typical metacentric- submetacentric pattern [17]. The morphologic similarity among sex chromosomes found in the *Leporinus* species led to the hypothesis of a common origin, in which an initial accumulation of heterochromatic segments was the first step in the differentiation of sex chromosomes [18]. An exception was found in *Leporinus* sp2, where the size of the W chromosome was similar to other elements of the karyotype [19]. These authors proposed that the sex chromosomal system in *Leporinus* sp2 originated independently from the ZW system previously described for other *Leporinus* species.

Our hybridisation experiments show that WLe hybridizes solely to the W chromosome in *L. elongatus*, while in *L. macrocephalus* and *L. obtusidens* the Z chromosomes were also partly labelled. The WLm and WLo probes reveal a similar result in cross species experiments, the most intense signals being those located at heterochromatic regions of the q arms. This pattern is possibly due the distribution of repetitive elements.

The use of repetitive elements to map the dynamics of sex chromosomes in Anostomidae has been successfully employed. The repetitive element Le*Spe*I was described as a participant of the sex chromosome differentiation process in *L. elongatus*[15]. This sequence was also mapped in *L. macrocephalus* and *L. obtusidens*, revealing homologies and highlighting their importance in sex chromosome differentiation [20]. However, the attempt to find a relationship between Le*Spe*I and a putative cryptic sex differentiation process in *Leporinus friderici, L. striatus*, *L. lacustris*, *Schizodon borelii* and *S. isognathus* by mapping this sequence in these species without sex chromosomes, failed because no positive hybridisation signals were observed [21].

The *Leporinus* genus has been divided hypothetically into two different groups according to cytogenetic and molecular records [18,22]. Our data reinforce the idea of the subdivision of *Leporinus* genus in at least two lineages: one comprising species with differentiated sex chromosomes and the other without sex chromosome differentiation. The W specific probes retrieved from *L. elongatus*, *L. macrocephalus* and *L. obtusidens* systematically cross hybridised with other W chromosomes, showing the similarity of these sequences. Based on this fact, it is possible to hypothesize that the sex chromosomes of *L. elongatus*, *L. macrocephalus* and *L. obtusidens* had a common origin. According to our results, the hypothesis of cryptic sex chromosomes sharing *Leporinus* W sequences in the other anostomids (*Leporinus friderici, L. striatus*, *L. lacustris*, *Schizodon borelii* and *S. isognathus*) can be discarded, consistent with previous studies on repetitive sequences. Furthermore, those sex chromosomes could have originated from the same autosomes, but in a different process of differentiation.

A fact to note in our results is that the probes of the W chromosome from *L. elongatus* paint the Z chromosome of *L. macrocephalus* males and females and the Z chromosome of *L. obtusidens* male but do not paint the *L. elongatus* Z chromosome present in males and females. This reinforces the hypothesis proposed by Parise-maltempi et al. [15] about the presence of a new system of multiple sex chromosomes in *L. elongatus.* The sex chromosomes of *L. elongatus* are probably in a different stage of differentiation from those of the other *Leporinus* species in which repetitive sequences shared between the Z and W chromosomes are not shared in *L. elongatus* chromosome Z.

Cross-species chromosome painting is a powerful tool to delineate syntenic chromosome regions among closely and distantly related species and to reconstruct chromosomal phylogeny [23-25]. In fish specifically, studies have brought new insights into the evolution of sex chromosome systems, and showed that in many cases, closely related groups have taken quite different evolutionary paths [9,10,26-29]. As in *Leporinus* species analysed in this report, some other fish groups also share a common origin of sex chromosomes [12,30-32].

Our data indicate that in spite of the often reported high plasticity of sex determination mechanisms in fish, in the Anostomidae family, and especially in some species of the *Leporinus* genus, the sex systems seem to be conserved and derived from a common ancestral pair. Probably the sex chromosomes are actually different, but the different W chromosomes could have been invaded with the same repetitive sequences. The results shown here add new data to studies using the *Leporinus* genus as a model, reinforcing the hypothesis that the *Leporinus* ZW sex chromosomes may be found only in closely related species, and remain undetected in the other species.

## Methods

### Cell culture and metaphase cell preparation

*Leporinus elongatus* chromosomes were obtained from cultured cells. Fibroblast cells collected from fin tissue of *L. elongatus* were cultured in DMEM medium supplemented with 10% FCS and 50% Amniomax C-100 (Invitrogen) serum at 37°C.

The cells were harvested after colcemid treatment (0.05 ug/mL) for 2 hours, suspended in hypotonic solution (0.075M KCl) and then incubated at 37°C for 20 min. Cells were pelleted and resuspended three times in fresh ice cold fixative (3:1 methanol:acetic acid), then kept at −20°C until use.

The chromosomes of other Anostomidae species (*L. friderici, L. striatus*, *L. lacustris*, *Schizodon borelii* and *S. isognathus*) were obtained by direct cytological preparations from kidney samples according to the methods given by Foresti et al. [33].

### Chromosome microdissection and DNA amplification

Chromosome suspensions were dropped onto moist, 1% SDS- cleaned coverslips. The coverslips were washed in 1x PBS solution for 1 minute and incubated in a trypsin solution (35 mL 1x PBS and 5 mL trypsin) for 30 seconds, washed again in 1x PBS and stained with Giemsa 1% in PBS.

The microdissection was performed using an Eppendorf TransferMan NK2 (Eppendorf) coupled to a Zeiss Axiovert 40 CFL microscope, with glass needles made with a Nikon puller and sterilized by UV radiation. About 8 chromosomes were separately placed in 9uL DNAse-free ultrapure water and were then amplified using the GenomePlex Single Cell Whole Genome Amplification Kit (WGA4-Sigma).

### Fluorescent *in situ* hybridisation (FISH)

The probes were labelled using the GenomePlex (WGA3 Reamplification KIT-Sigma) following the manufacturer’s protocol, except changing the kit dNTP mix for a ½ T dNTP mix and adding a biotin 16-dUTP in the reaction.

Fluorescent in situ hybridisation (FISH) was performed using the protocol described by Rens et al. [34,35] with several modifications.

Slides were dehydrated through an ethanol series; aged for 1h at 60°C, denatured in 70% formamide/0.6x saline-sodium citrate (SSC) at 65°C for 2 minutes and dehydrated again. 3 μL of biotinylated probe combined with *L. elongatus Cot* 1 (2 μg) was precipitated in ethanol and resuspended in 13 μL of hybridisation buffer (40% deionized formamide (v/v), 10% dextran sulfate, 2x SSC, 0.05 M phosphate buffer, pH 7.3). This mixture was denatured for 10 min at 65°C, pre-annealed at 37°C for 1 hour and applied to each slide. Hybridisation was carried out at 37°C for 12 hours for the same species and over three nights for cross species experiments. It was used Cot1 once that Fish genomes possess a high amount of repetitive elements and FISH with chromosome specific probes gives a large amount of background signal if no suppression of repetitive elements is used. Post-hybridisation washes were performed in 40% formamide/1.8x SSC twice for 5 min each, followed by 2x SSC twice for 5 min each and 4x SSC with 0.05% Tween-20 (4xT) once for 4 min at 42°C for the same species and 45°C for cross species. Probe detection was carried out using 200 μL of diluted (1:500) Cy3-streptavidin antibody (Amersham) per slide at 37°C for 30 min. After detection, slides were washed in 4xT three times for 3 min each at 42°C and mounted in Vectashield mounting medium with 4′,6- diamidino-2-phenylindole (DAPI; Vector Laboratories).

Images were captured and processed using the CytoVision Genus system (Applied Imaging, USA) and a Cohu CCD camera mounted on an Olympus BX-60 microscope.

## Competing interests

The authors declare that they have no competing interests.

## Authors’ contributions

PPPM, FD, WR conceived and designed the experiments. PPPM and ELS performed the experiments and drafted the manuscript. WR and FD helped in cell culture and with developing the laboratory techniques. PCMO gave important suggestions about cell culture and FISH analysis. VT made substantial contributions in microdissection experiments. Most of the experiments were performed in MAFS laboratory. All authors read and approved the final manuscript.
